# Impact of the L-arginine-Nitric Oxide Pathway and Oxidative Stress on the Pathogenesis of the Metabolic Syndrome

**DOI:** 10.2174/1874091X00802010108

**Published:** 2008-07-14

**Authors:** C.R Assumpção, T.M.C Brunini, C Matsuura, A.C Resende, A.C Mendes-Ribeiro

**Affiliations:** Departamento de Farmacologia e Psicobiologia, Instituto de Biologia, Av. 28 de Setembro 87 CEP 20551-030, Rio de Janeiro, Brazil

**Keywords:** Nitric oxide, L-arginine, metabolic syndrome, blood cells, endothelium.

## Abstract

The discovery of the physiological roles of nitric oxide has revolutionized the understanding of regulation of vascular tone, platelet adhesion and aggregation, and immune activation. Perhaps the most intriguing aspect of nitric oxide (NO) is that it is a gas that, in the absence of receptors, can regulate both normal physiological events and mediate cytotoxicity under pathological conditions. NO is produced from L-arginine by NO synthases (NOS), yielding L-citrulline and NO. The regulation of L-arginine pathway activity occurs at the level of NO production. The metabolic syndrome is a cluster of insulin resistance, elevated blood pressure, and atherogenic dyslipidemia, a common basis of cardiovascular disease. It occurs in genetically susceptible individuals with environmental influences and has serious economic and social consequences. Pharmacological and non-pharmacological therapies should be individualized and targeted to normalize its alterations of blood pressure, HDL cholesterol, triglycerides and glucose values. Despite the increasing prevalence of the metabolic syndrome in the last decades, there has been little progress in the understanding of the precise mechanisms involved in the pathogenesis of this syndrome and its complications. Emerging evidence is available that NO, inflammation and oxidative stress play important roles in the physiopathology of this syndrome. This review summarizes and evaluates the participation of the L-arginine-NO pathway and oxidative stress in the physiopathology of the metabolic syndrome and cardiovascular events at the systemic level, as well as the effects of exercise on this syndrome.

## 1. INTRODUCTION

Metabolic syndrome is characterised by glucose intolerance, insulin resistance, abdominal adiposity, elevated blood pressure, and dyslipidaemia and is associated with increased morbidity and mortality, due at least in part to vascular complications [1, 2].

Recently, studies in animals and humans have suggested that endothelial-nitric oxide (NO)-mediated vasorelaxation is impaired and decreases the blood flow to skeletal muscle in metabolic syndrome [2]. NO exerts important vasodilatory, antiplatelet, antioxidant, antiadhesive and antiproliferative effects [3]. Inappropriate release of this mediator or impaired availability of its precursor L-arginine may contribute to the development of clinically significant atherosclerosis and increase the tendency for thrombus formation in this syndrome [2].

In the present study, we review the current state of knowledge regarding the role of NO, endothelial dysfunction and oxidative stress in metabolic syndrome and the effects of exercise, a non-pharmacological tool.

## 2. METABOLIC SYNDROME: DEFINITION AND EPIDEMIOLOGY

The concept of metabolic syndrome has existed for at least 80 years. In the 1920s by Kylin, a Swedish physician, as the clustering of hypertension, hyperglycaemia, and gout. Later, in 1947, Vague drew attention to upper body adiposity (android or male-type obesity) as the obesity phenotype that was commonly associated with metabolic abnormalities occurring in type 2 diabetes and cardiovascular disease [4]. In 1988, Reaven described syndrome X, which includes insulin resistance, hyperglycaemia, hypertension, low HDL-cholesterol, and raised VLDL-triglycerides [5]. This syndrome is an intermediate state between normal metabolism and type 2 diabetes mellitus, and was subsequently called metabolic syndrome [6].

The third report of the US National Cholesterol Education Program (NCEP) Adult Treatment Panel III (ATP III) has provided a definition of the metabolic syndrome to aid in the diagnosis and treatment of patients at risk for chronic heart disease (CHD). The NCEP ATP III criteria require ≥ 3 of the 5 risk factors listed in Table **1** [1].

Eckel *et al*. [4] related that prevalence of metabolic syndrome is very age-dependent but is high in obese children and adolescents, and increases with worsening obesity. The prevalence of MS varies in urban populations from 8% (India) to 24% (USA) in men, and from 7% (France) to 43% (Iran) in women [4].

The NCEP ATP III suggests that metabolic syndrome is associated with the development of CHD. However, the level of risk associated with the syndrome is unclear. In a Finnish population-based, prospective cohort study, the risk for cardiovascular disease mortality over a 12-year follow-up period was clearly higher in subjects with metabolic syndrome versus those without [7]. The unadjusted RR of cardiovascular disease mortality was high at 3.55, suggesting that patients with metabolic syndrome may have as high a risk for cardiovascular disease mortality as patients with prior CHD [7].

## 3. L-ARGININE TRANSPORT AND NITRIC OXIDE PRODUCTION

**Fig. (1). L-arginine-nitric oxide pathway.**
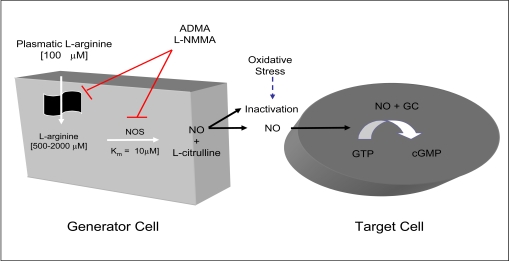
NO=nitric oxide; NOS=nitric oxide synthase; GC=guanylase cyclase; GTP=guanosine triphosphate; cGMP=cyclic guanosine monophos-phate; ADMA=asymmetric dimethylarginine; L-NMMA.

NO is generated, in mammalian cells, from the amino acid L-arginine using the enzyme nitric oxide synthase (NOS), with the same spectral characteristic as the cytochrome P-450 family and an ED50 for L-arginine of about 6 µM [3, 8-10]. NO synthases include four prosthetic sets in their structure: flavin-adenine dinucleotide (FAD); flavin mononucleotide (FMN); tetrahydrobiopterin (H4 biopterin) and a haeme complex. The production of nitric oxide from L-arginine involves two monooxygenation steps requiring O2 and NADPH: an initial hydroxylation of L-arginine to generate N-hydroxyarginine and an oxidation leading to the formation of NO and citrulline [3, 8-10] (Fig. **1**).

In mammals, all NOS derive from three genes. The three isoforms (neuronal) nNOS, (inducible) iNOS and (endothelial) eNOS are differentiated according to their location, dependence on increased cytosolic Ca2+ for their activity (> ~ 100 nM), duration of action and the fact that iNOS is inducible while the other two are constitutive [3, 8] (Table **2**). Recently, a NOS located in the mitochondria (mtNOS) has been observed in different brain regions, which is responsible for the production of NO in these organelles and identified as nNOS [11].

Nitric oxide is a potent endogenous vasodilator that prevents platelet adhesion and aggregation and inhibits vascular smooth muscle proliferation. Platelets themselves also possess nitric oxide synthases whose detailed structure has been identified [13].

The intracellular concentration of L-arginine is usually well above the Km for nitric oxide production (~3-4 mM in endothelial cells, macrophages and smooth muscle cells) [14]. However, under certain conditions, e.g. when endothelial cells are placed in an L-arginine-free medium [15] or when L-arginine transport is inhibited with antisense nucleotides to the transporter [16], NO production is diminished.

Humans synthesise L-arginine analogues, such as asymmetric dimethylarginine (ADMA) and monomethyl-L-arginine (L-NMMA), which are endogenous inhibitors of NOS and L-arginine transport that enhance platelet aggregation and increase blood pressure [17].

NO activates soluble guanylate cyclase in the target tissue binding to heme protoporphyrin to produce cGMP, which in turn phosphorylates cGMP-dependent protein kinase (PKG). PKG acts at several sites within the cell membrane and sarcoplasmic reticulum lowering intracellular Ca^2+^ levels ([Ca^2+^]i), dephosphorylaing myosin light chain, and also decreasing its Ca^2+^ sensitivity [18]. As a result, there is a decrease vascular tone, since the phosphorylation of Ser-19 on the 20-kDa myosin regulatory light chains is the primary determinant of cross-bridge attachment and cycling during contraction and relaxation in smooth muscle [19]. The [Ca^2+^]i decrease mediated by PKG can be explained by: (1) activation of PKG-dependent K^+^-channel [20] and Ca^2+^-dependent K^+^-channels [21] resulting in membrane hyperpolarization and inhibition of Ca^2+^ entry through voltage-gated Ca^2+^-channels in the cell membrane[22]; and (2) increased cellular extrusion of Ca^2+^ *via *activation of Ca^2+^-ATPAse pumps and Na^+^/Ca^2+^ exchanger[23,24].

Conversely, [Ca^2+^]i can also be reduced by an increase in cytosolic Ca^2+^ uptake *via *sarcoplasmic reticulum Ca^2+^ATPase (SERCA) activation, a cGMP-independent pathway [25]. However, this action is not mediated by NO itself, but rather, by peroxynitrite after NO reaction with superoxide in physiological concentrations. SERCA is activated by reversible S-glutathiolation by peroxynitrite [26]. Thus, the response to authentic NO depends upon both cGMP-dependent inhibition of contractile proteins and cGMP-dependent and -independent decrease in intracellular free calcium levels that act cooperatively to relax smooth muscle and mediate vasodilation.

## 4. THE PATHOPHYSIOLOGY OF METABOLIC SYNDROME: THE ROLE OF L-ARGININE-NITRIC OXIDE PATHWAY

**Fig. (2).Effects of metabolic syndrome in nitric oxide bioavailability.**
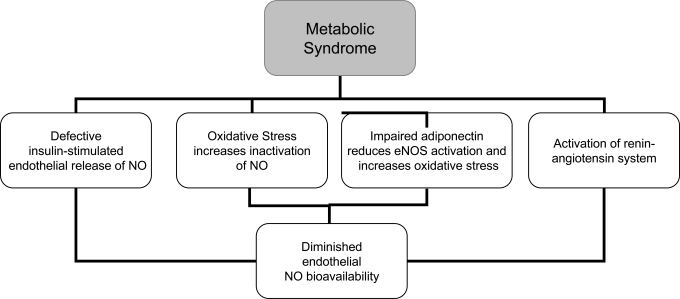
NO=nitric oxide; eNOS= endothelial nitric oxide synthase.

The pathophysiology of the metabolic syndrome remains a subject of continuing controversy. There are studies suggesting that insulin resistance is of central importance in this syndrome. One third of an apparently healthy population is sufficiently insulin resistant to develop significant clinical disease [27, 28]. Insulin resistance has traditionally been defined from a glucocentric view—i.e., when a defect in insulin action results in fasting hyperinsulinaemia to maintain euglycaemia [4]. Insulin has important vascular actions to stimulate production of NO in endothelium, leading to increased blood flow that contributes significantly to insulin-mediated glucose uptake. Insulin signaling pathways in the vascular endothelium regulating production of NO share striking similarities with metabolic insulin signaling pathways in skeletal muscle and adipose tissue [29]. Defective insulin-stimulated endothelial release of NO appears to be responsible, in part, for impaired capillary network expansion and the inability of insulin to redirect blood flow in the microcirculation towards metabolically active tissues (Fig. **2**). As a result, the diffusion of insulin and its metabolic substrates is delayed and diminished, further aggravating the underlying insulin resistance. Moreover, the major factor in determining the rate of insulin transport across the capillary bed must be the size of the capillary bed itself or the inability of insulin to recruit previously closed capillary beds. In addition to insulin, adipocytokines and other endothelial products have been identified in insulin resistant obese individuals that are capable of altering capillary permeability, and thus could also interfere with the insulin’s metabolic action [30].

In target tissues, insulin stimulates two major pathways: the phosphatidylinositol 3–kinase pathway and the mitogen-activated protein kinase (MAPK) pathway. The MAPK pathway has been shown also to regulate insulin-dependent endothelial NO production. In the presence of a defect in insulin-mediated glucose uptake, it has been demonstrated that there is also a defect in insulin-stimulated endothelial vasodilation. Thus, a systemic defect in the phosphatidylinositol 3–kinase pathway, which likely defines insulin resistance, leads to a combined defect in insulin-mediated glucose transport and in insulin stimulated endothelial vasodilation [31].

A study by Michio Shimabukuro *et al*. [32] demonstrated in islets of rats predisposed to NIDDM (non–insulin-dependent diabetes mellitus) that long-chain fatty acids influence pancreatic *β *cells *via *the NO system. In other tissues, NO is thought to have a dual role, serving as a regulator under physiologic conditions and as a cytotoxin under pathophysiologic ones. The cytotoxic role of NO can be induced by FFA in islets of rats predisposed to NIDDM. It is possible that the higher NO levels in islets of Zucker Diabetic Fatty (ZDF) rats result in greater production of toxic hydroxyl ions from peroxynitrite.

Microvascular dysfunction is a cardinal feature of the metabolic syndrome that affects pressure and flow patterns, increasing peripheral vascular resistance and decreasing sensibility for insulin-mediated glucose disposal, contributing to hypertension and insulin resistance, respectively [33]. In obese Zucker rats, an animal model of the metabolic syndrome, reduced endothelium-mediated dilation was associated with decreased NO bioavailability and excessive superoxide production [34]. Moreover, reduced skeletal muscle microvessel density (MVD) in this animal model seems to be a function of a chronic vascular reduction of NO [35]. In another animal model of the metabolic syndrome, chronic consumption of a high-fat diet by Fischer rats, the presence of endothelial dysfunction and oxidative stress, which can diminish NO bioavailability, was confirmed [36].

In patients with metabolic syndrome, responses to intra-brachial acetylcholine were attenuated with no difference between normotensive and hypertensive groups [37]. NO level correlates with body mass index, systolic blood pressure and triglycerides in these patients [38].

Studies indicate that vasodilatation, *per se*, does not increase muscle glucose uptake. However, when vasodilatation occurs concomitantly with the recruitment of new capillary beds, as brought about by insulin, muscle glucose uptake is enhanced. In the same way that insulin resistance may contribute to endothelial dysfunction, defects in NO-mediated vasodilation may contribute to insulin resistance [29, 30, 39].

Increased levels of ADMA, a competitive inhibitor of L-arginine metabolism by transport and NO synthase, are associated with endothelial dysfunction and increased cardiovascular risk in various diseases [40]. Plasma levels of ADMA were positively correlated with insulin resistance in non-diabetic, normotensive people [41]. However, ADMA was not found to be elevated in subjects with metabolic syndrome. Furthermore, no significant association between ADMA concentrations and the degree of insulin resistance was found in this group of patients [42].

Adiponectin may exert some of its insulino-mimetic actions by stimulating phosphorylation and activation of eNOS in vascular endothelium, resulting in increased production of NO. Thus the novel role of adiponectin in eNOS activation, which explains both the metabolic and anti-atherogenic properties of adiponectin.[29].

Activation of the renin-angiotensin-aldosterone system (RAS)and subsequent elevations in angiotensin II and aldosterone,as seen in the metabolic syndrome, also contribute to altered insulin/IGF-1signaling pathways and reactive oxygen species formation toinduce endothelial dysfunction and cardiovascular disease. Several components of the RAS such as angiotensinogen, ACE (angiotensin-converting-enzyme), and angiotensin type 1 (AT1) receptors are present within human adipose tissue. Experimental studies suggest that the adipose RAS is regulated by hormonal and nutritional factors and correlates with the degree of obesity and that AII may modulate adipose tissue blood flow, growth, and metabolism. Clinical trials with ACE inhibitors and Angiotensin Receptor Blockers (ARB) suggest that inhibition of the RAS alters glucose metabolism in humans. Thus, endothelial dysfunction can be reversed by short and long-term AT1 receptor blockade. This cardioprotective effect is associated with increased NO bioavailability [6].

It has also been demonstrated that agents which improve endothelial function, an angiotensin-converting enzyme inhibitor and a statin, not only slowed the progression of CAD and occurrence of cardiovascular death but reduced the onset of type 2 diabetes in high-risk patients by approximately 30% and 35%, respectively [31].

The thiazolidinediones are peroxisome proliferator-activated receptor–γ agonists that improve glucose and lipid metabolism. These agents have recently been shown to improve endothelial function in the early stages of insulin resistance [31].

Aggressive pharmacological treatment of dyslipidemia and hypertension, even before the onset of MS, would appear prudent in decreasing the progression of the atherosclerotic process [31]. So this can be the relationship among the molecular basis, insulin resistance, obesity, atherosclerosis and other vascular complications of diabetes present in the MS that need treatment.

Moreover,consumption of a Mediterranean-style diet by patients with the metabolic syndrome was associated with improvement of endothelial function and a significant reduction in markers of systemic vascular inflammation [43].

## 5. METABOLIC SYNDROME AND OXIDATIVE STRESS

Over the past decades, a great deal of research has been carried out to seek an understanding of the role of oxygen toxicity as well as reactive oxygen species (ROS) in a wide variety of pathological conditions [44]. A good deal of evidence has emerged which demonstrates a close link among the metabolic syndrome, a state of chronic low-level inflammation and oxidative stress as second-level abnormalities [45]. Experimental and clinical observations indicate oxidative stress as an important mechanism in hypertension, diabetes and obesity-associated metabolic syndrome and its complications [46, 47]. Excessive free radical production and oxidative damages appear to explain, at least in part, the perpetuation of insulin resistance, altered energy production, endothelial dysfunction and the appearance of vascular complications in this condition.

A number of clinical studies have reported the importance of visceral fat accumulation in the development of metabolic disorders, including reduced glucose tolerance, hyperlipidemia and cardiovascular diseases. It is generally accepted that the sequence of events leading to hepatocyte fatty degeneration begins with insulin resistance, which precedes fat accumulation [48]. Excess intracellular fatty acids, oxidative stress, energy depletion and mitochondrial dysfunction then cause cellular injury [48-50].

Hyperglycemia is the fundamental abnormality underlying the mechanisms causing endothelial dysfunction in diabetes. In fact, hyperglycemia-induced ROS formation may successively lead to endothelial dysfunction by decreasing NO and prostacyclin bioavailability and by increasing the synthesis of vasoconstrictor prostanoids and endothelin [51]. In addition, the early and high incidence of atherosclerosis and cardiovascular events in patients with diabetes and postprandial high blood glucose levels has been partially associated with oxidative stress [52]. The inflammation associated with the atherosclerotic process is modulated by the activity of several families of enzymes, including cyclooxygenases, lipoxygenases, Nox, NO synthases and peroxidases — all possessing the capacity to produce ROS and NO species.

The participation of arterial hypertension in the generation of systemic oxidative stress associated with the metabolic syndrome is suggested by a number of observations of the NO metabolism changes and the low circulating levels of vitamin C in patients with high-grade hypertension [53] and the improvement of systemic oxidative stress with antihypertensive treatment [54]. Elevated levels of superoxide anion (O^-^_2_) and peroxinitrite (H_2_O_2_) are present in essential hypertensive patients [55, 56]. Moreover, reduced antioxidant defence mechanisms such as superoxide dismutase (SOD), catalase and glutathione peroxidase have been reported in blood cells and vessels from hypertensive patients and experimental hypertension [55, 57, 58]. These considerations have an even higher impact when associated with endothelium activation and dysfunction as characterised by increased levels of circulating oxidised LDL, intercellular and vascular adhesion molecules and C-reactive protein [59], and with the evidence that vascular complications are also associated with oxidative stress events [60].

Recent proposed mechanisms for hypertension development in metabolic syndrome, attribute a central role to insulin resistance and oxidative stress. It has been demonstrated that visceral adipose tissue produces and secrets a variety of bioactivity substances termed adipocytokines, such as leptin, tumor necrosis factor-α (TNF-α), interleukin-6 (IL-6), angiotensin II and non-esterified fatty acids (NEFA), which induce insulin resistance, oxidative stress and development of hypertension [61]. Insulin resistance seems to be the main pathophysiologic feature of the metabolic syndrome and some mechanisms connect insulin resistance with hypertension in this syndrome. The anti-natriuretic effect of insulin may be increased in individuals with insulin resistance and this effect may play an important role for development of hypertension. *In vitro* studies have shown that insulin stimulates both endothelin-1 production and its action on the vascular wall and induction of ROS. The renin-angiotensin system (RAS) plays a crucial role in blood pressure regulation, by affecting function and by modulating vascular tone [62]. The activity of the RAS appears to be regulated by food intake, and overfeeding of rodents has been reported to lead to increased formation of angiotensin II in adipocytes [63]. Therefore, the alterations induced by insulin resistance may be amplified by increased angiotensin II levels that induce vasoconstriction, sodium retention, generation of ROS, reduced availability of NO and vascular damage inducing hypertension.

Relatively new and interesting pathways of oxidative stress-induced vascular damages include enzymes such as Nox and homocysteine [64, 65]. Membrane-bound Nox is a major source of ROS in preatherosclerotic conditions and has been found in human peripheral and coronary arteries [66, 67]. Enhanced expression and activity of Nox enzymes have also been detected in new accumulated adipose tissue of obese mice and have been related to impaired antioxidant defence and adipocytokine dysregulation [46]. By increasing oxidative stress, activation of Nox in vascular cells has been reported to be an important mechanism in the pathogenesis of hypertension and atherosclerosis [68]. Angiotensin II is one of the most potent stimuli activating vascular Nox. This property clearly links ROS production with activation of the renin–angiotensin system in hypertension [69]. As a consequence, drugs acting on the renin–angiotensin system reduce Nox activity, thus rendering this enzyme a specific drug target.

## 6. EXERCISE AS A NON-PHARMACOLOGICAL THERAPY IN THE TREATMENT OF METABOLIC SYNDROME

Lifestyle changes, including an increase in physical activity, are recommended for the treatment of metabolic syndrome [70]. There is an extensive body of knowledge regarding the beneficial effects of physical activity on metabolic risk factors and atherosclerotic cardiovascular disease [71]. Exercise training, especially endurance exercise, has been shown to decrease body mass and visceral fat accumulation, improve insulin sensitivity, and decrease triglyceride levels and systolic and diastolic blood pressure [72, 73]. Higher plasma levels of HDL cholesterol after exercise training is not a consistent finding, since some studies show a decrease in these levels in metabolic syndrome patients. However, it is important to highlight that there is an improvement in the protective capacity of HDL against LDL oxidation [74], and a reduction in the LDL/HDL ratio [75]. These favourable changes can occur independent of changes in dietary energy intake [76]. Besides exerting positive effects on its individual features, exercise training can either prevent or treat metabolic syndrome itself. The prevalence of this syndrome is lower among those with higher physical activity and fitness level [77], and it was observed that after 20 weeks of endurance training, 30.5% of 105 subjects were no longer classified as having metabolic syndrome in the HERITAGE Family Study [72].

In animal model studies, moderate intensity endurance training has been shown to enhance endothelium-dependent vasodilation *via *NO, since the enhanced relaxations to ACh were abolished by L-NAME, an L-arginine analogue that inhibits NOS, but not by diclofenac, an inhibitor of cyclooxygenase, and preconstriction with KCl [78, 79]. Endurance training also seems to be effective in preventing the reduction of microvessel density in the exercising muscle resulting from improved NO bioavailability and an altered profile of inflammatory markers associated with angiogenesis, such as monocyte chemoattractant protein-1 and IL-1β [80].

Similar findings were observed in humans [74]. Metabolic syndrome patients were submitted to daily endurance training at 70 to 85% of the maximal heart rate combined with a high-fibre, low-fat diet. After 3 weeks of training, besides an improvement in the lipid and metabolic profile, there was a decrease in oxidative stress, assessed by serum measurements of 8-isoprostaglandin F_2α_ and myeloperoxidase, and an increase in NO production in cultured human aortic endothelial cells. Exercise and a high-fibre, low-fat diet also resulted in reduced inflammation, endothelial cell and platelet activation, monocyte adhesion and monocyte-chemotactic activity, and MMP-9, a marker of plaque desestabilization. One interesting finding was that this improvement occurred independent of weight loss.

Endurance exercise has also been shown to improve insulin sensitivity in humans [73] and animals [81]. Following 2 weeks of exercise training, obese Zucker rats presented an enhancement of insulin action on skeletal muscle glucose transport activity, primarily in the exercised musculature [81]. The mechanisms responsible for this change are still unknown, although training induces an increase in GLUT 4 protein expression and in the activity of enzymes involved in glucose metabolism, such as hexokinase and citrate synthase [81]. Exercise also augments insulin-mediated glucose transport in skeletal muscle and the expression of insulin receptor substrate (IRS)-1 protein and IRS-1-p85 interaction, suggesting an improvement in the insulin stimulation of the IRS-1/PI 3-kinase pathway.

Part of these changes may also be attributed to the low levels of adiponectin in metabolic syndrome patients, the most abundant adipokine secreted by adipocytes. Adiponectin acts through two receptors, AdipoR1 and AdipoR2, which are primarily expressed in skeletal muscle and the liver, respectively [82], and both are also present in endothelial cells [83, 84]. Chang *et al*. [85] demonstrated that 8 weeks of aerobic exercise increased the mRNA and protein content of AdipoR1 in the exercised skeletal muscle of OZR, associated with an improvement in insulin sensitivity, assessed by a glucose-insulin index (the product of the areas under the curve of glucose and insulin) during an intraperitoneal glucose tolerance test. This is an important finding, since adiponectin is involved in the sensitivity modulation by stimulating glucose utilisation and fatty acid oxidation *via *the phosphorylation and activation of AMPK in both muscle and liver cells [86, 87]. It is important to highlight that adiponectin has also emerged as a cardioprotective adipokine that possesses antiatherogenic, and anti-inflammatory properties due to NO bioavailability modulation. *In vitro*, adiponectin induces NO production in human aortic endothelial cells *via *activation of the AMPK pathway and enhanced endothelial eNOS RNA protein expression [88, 89]. Moreover, adiponectin suppresses superoxide generation and enhances eNOS activity in endothelial cells treated with oxidised LDL [83].

When thinking of a "cardioprotective" polypill as suggested by Wald and Law in 2003 [90], it is not possible to ignore the important role that physical activity plays in different cardiovascular risk factors. Although the precise mechanisms responsible for its benefits are still largely unknown, endurance training was shown to reduce circulating levels of inflammatory markers and oxidative stress, and increase NO bioavailability

## 7. CONCLUSION

Although comprehension of the complex multiorgan system derangements that compose the metabolic syndrome has improved over the years, a reasonable understanding of the defects in specific cellular and sub-cellular functions has not been completely attained. Studies have indicated that metabolic syndrome is characterised by abnormal regulation of NO synthesis by pancreatic and adipocytes cells and at the systemic level associated with increased oxidative stress. Growing evidence suggests that endurance exercise, a non-pharmacological tool, can have a favourable impact on the metabolic alterations by reducing circulating levels of inflammatory markers and oxidative stress, and increasing NO bioavailability, therefore diminishing the risk of any cardiovascular event.

## Figures and Tables

**Table 1.** The National Cholesterol Education Program's Adult Treatment Panel III (NCEP: ATP III , 2001)

NCEP: ATP III,2001
3 or more of the following:
Central obesity: waist circumference >102 cm (male), >88 cm (female)
Hypertriglyceridaemia: triglycerides ≥1·7 mmol/L
Low HDL cholesterol: <1·0 mmol/L (male), <1·3 mmol/L (female)
Hypertension: blood pressure ≥130/85 mm Hg or medication
Fasting plasma glucose ≥6·1 mmol/L

**Table 2.** Summary of the Features of the Three NOS Isoforms

	**nNOS or type I**	**iNOS or type II**	**eNOS or type III**
**First identification**	neurones	macrophages	Endothelium
**Molecular weight (kDa)**	160	130	133
**Cromossomal localisation**	12p24.2	17cen-q12	7q35-36
**Major function**	Neuronal messenger	Immunocytotoxicity	Relaxation of VSM^*^
**Levels of NO produced**	pmoles	nmoles	pmoles
**Regulation of expression**	Constitutive Up-regulated by sex hormones, nerve and brain injury	Not normally present Expression induced by cytokines and endotoxin	Constitutive Up-regulated by sex hormones and shear stress

^*^VSM=vascular smooth muscle. Refs. [3, 8-10, 12].
